# A commentary on: TDO2‐augmented fibroblasts secrete EVs enriched in immunomodulatory Y‐derived small RNA

**DOI:** 10.1002/jex2.99

**Published:** 2023-08-18

**Authors:** Clarissa Sastrawidjaya, Phuong H. D. Nguyen

**Affiliations:** ^1^ Department of Pharmacology, Institute for Digital Medicine National University of Singapore Singapore Singapore

**Keywords:** extracellular vesicles, tryptophan 2,3‐dioxygenase, fibroblasts, myocardial infarction, regenerative medicine

Extracellular vesicles (EVs) containing biological information in the form of proteins, lipids, and nucleic acids can be shuttled between cells to mediate physiological responses (Raposo & Stahl, 2019). Functions of EVs in healthy states include intercellular communication, cell survival, angiogenesis, inflammatory and immune response, coagulation, and waste management (Yuana et al., 2013). By the same token, EVs are involved in pathophysiological conditions. Alterations in EV cargo following changes to the pathophysiological state of donor cells correlate with disease progression (Saheera et al., 2021). EVs have attracted immense interest in the field of cell‐free therapeutics. Compared to cells, EVs are more stable, less immunogenic, and can tolerate repeated freeze–thaw cycles, making them less burdensome for manufacture and storage (Akers et al., 2016; Murphy et al., 2019; Usman et al., 2018). Due to their nanoscale dimensions, EVs are in some instancescapable of permeating into tissues and crossing cellular barriers (Banks et al., 2020; Saint‐Pol et al., 2020). They may therefore be more efficient in reaching target sites of action.

In the context of tissue injury, the ability of EVs to modulate the immune response suggests their therapeutic potential. Various studies have demonstrated the importance of EVs in restoring tissue homeostasis following acute injuries, trauma, surgeries, and chronic diseases (Arslan et al., 2013; Gallet et al., 2016; Kervadec et al., 2016; Xiong et al., 2020, 2017,2020, 2017). After all, immune responses crucially determine the prognosis of tissue injury, whereby outcomes range from fibrosis to complete regeneration depending on the type, duration, and cellular mediators underlying the immune response (Julier et al., 2017). The present commentary addresses the findings of Ciullo et al. that a small Y‐derived RNA, NT4, mediates immunomodulatory effects when enriched in EVs secreted by tryptophan 2,3‐dioxygenase (TDO2)‐upregulated fibroblasts (TDO2‐EVs) (Ciullo et al., 2023) (Figure 1). NT4 enrichment allows TDO2‐EVs to attenuate inflammatory profiles in macrophages, contributing to cardioprotection in disease states such as acute myocardial infarction (AMI).

**FIGURE 1:**
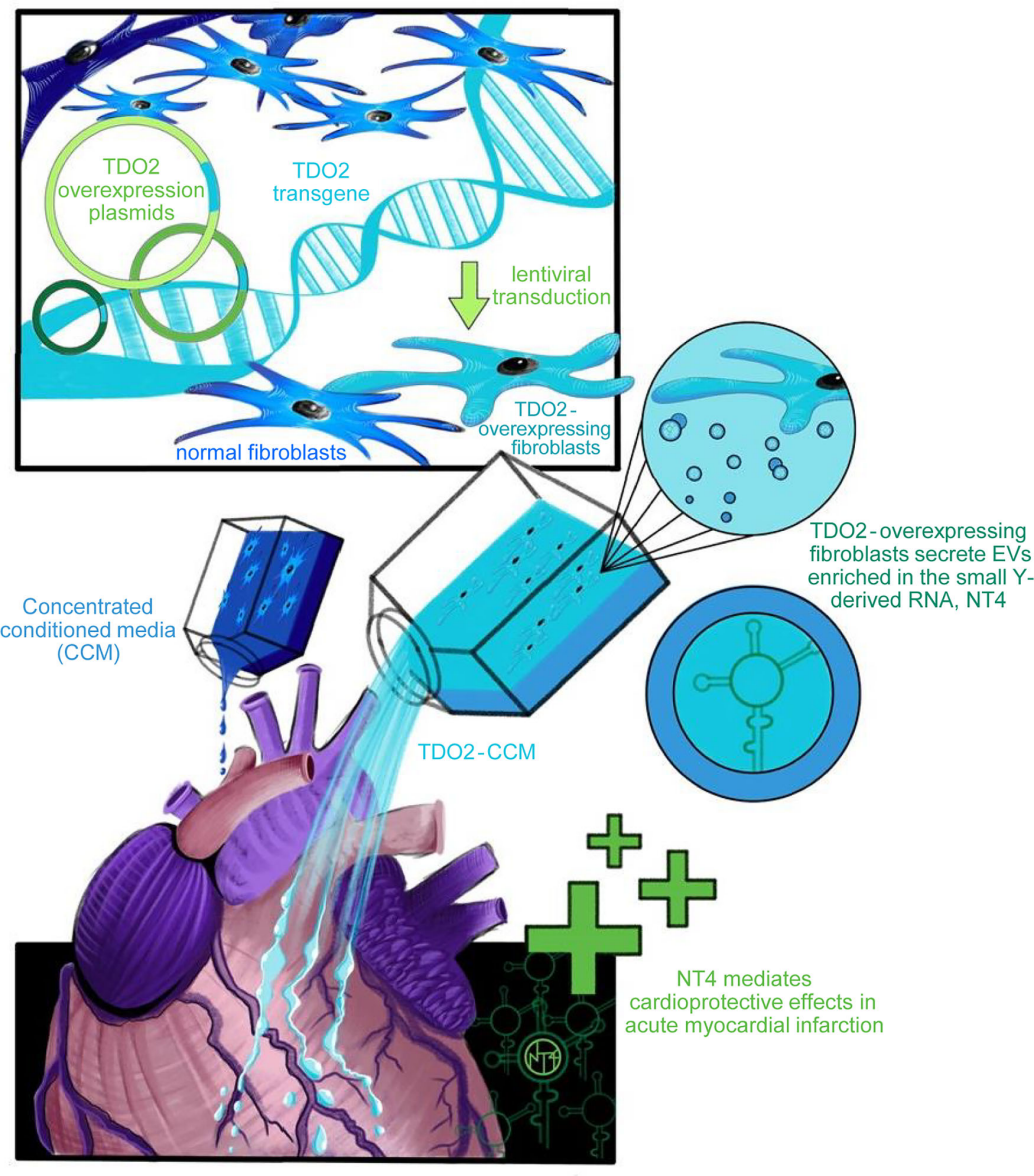
Upregulation of tryptophan 2,3‐dioxygenase (TDO2) in human dermal fibroblasts leads to secretion of extracellular vesicles with therapeutic potential. Inert human dermal fibroblasts (HDFs) were lentivirally transduced to overexpress TDO2. Extracellular vesicles (EVs) in the concentrated conditioned media (CCM) of TDO2‐upregulated fibroblasts (TDO2‐CCM) are enriched in NT4, a small Y‐derived RNA which exerts immunomodulatory effects. In comparison to CCM collected from wild‐type HDFs (HDF‐CCM), TDO2‐CCM was found to reduce cardiac tissue damage when administered to the mouse model of acute myocardial infarction by intramyocardial injection. The therapeutic effects of NT4 could potentially improve the prognosis of tissue injury from fibrosis to regeneration.

In myocardial infarction (MI), myocardial tissues undergo ischemic death, resulting in a large‐scale loss of cardiac muscle including cardiomyocytes, endothelial cells, and fibroblasts (Laflamme & Murry, 2005). Depending on the severity of the damage, patients may suffer from progressive contractile dysfunction. Following MI, a process of repair, known as ventricular remodelling, stimulates a vigorous inflammatory response to recruit immune cells to the injured site for clearing apoptotic cells and debris. However, unrestrained inflammation post‐MI may lead to further damage and autoimmunity. Previous studies have shown that cardiosphere‐derived cells (CDCs) and their EVs elicit cardioprotective effects by suppressing inflammatory response while promoting cellular renewal and angiogenic pathways (Geoffrey De Couto et al., 2019; Ibrahim et al., 2014). Additionally, Ahmed G. E. Ibrahim and colleagues found that activation of Wnt/β‐catenin signalling and the downstream target gene, TDO2, could induce therapeutic potential in CDCs and normal human dermal fibroblasts (Ibrahim et al., 2021, 2019,2021, 2019). Since CDCs and immortalized CDCs exhibit inconsistent potency across heathy donors and lots, genetically engineered normal human dermal fibroblasts could serve as an immortal source of therapeutically potent EVs (Ibrahim et al., 2019). These data present promising opportunities for regenerative medicine.

In a previous paper published by the same group, inert fibroblasts with enhanced expression of TDO2 were found to produce EVs with anti‐inflammatory capabilities (Peck et al., 2021).

Overexpression of TDO2 was induced in neonatal human dermal fibroblasts (nHDFs) by lentiviral transduction of the TDO2 transgene under control of a constitutive promoter (Peck et al., 2021). TDO2‐EVs were found to attenuate inflammatory signalling in macrophages by inducing an anergic phenotype via an NFкB‐dependent mechanism. The group then established in the same paper that these nHDF^TDO2^‐EVs exert a cardioprotective effect in an AMI model where macrophages play an important role in resolving cardiac injury (Peck et al., 2021). To better understand the mechanisms by which TDO2‐EVs suppress inflammation, the group sought here to identify precise determinants of therapeutic potency within EV payloads (Ciullo et al., 2023). Concentrated conditioned media (CCM) was first isolated from normal HDFs, alongside HDFs which had been lentivirally transduced to overexpress TDO2. The AMI mice administered with CCM from TDO2‐transduced cells (TDO2‐CCM) had reduced levels of circulating cardiac troponin I (cTnI) after 24 h, improved ejection fraction after three weeks, and showed a tendency towards lower end diastolic and systolic volumes. Reduced scar formation was also observed in infarcted hearts treated with TDO2‐CCM. In addition, RNA was isolated from the infarct and border zone tissue 24 h post‐injury. Transcriptomic analysis revealed the downregulation of pro‐inflammatory and pro‐senescent genes in TDO2‐CCM‐treated hearts.

Ciullo et al. mentioned that they used nanosight tracking analysis to determine size and concentration of the EVs (Ciullo et al., 2023). Although this work focuses on the mechanism by which TDO2‐CCM exerts cardioprotective effects, more in‐depth information about EV profiles is recommended to further examine the specific roles of EVs in this therapeutic platform. According to the Minimal Information for Studies of Extracellular Vesicles 2018 (MISEV2018), EVs of high purity are required for functional studies because co‐isolated contaminants may confound the EV functions observed (Thery et al., 2018). Furthermore, it is recommended by the International Society for Extracellular Vesicles to characterize EV samples for the enrichment of positive markers such as CD9, CD63, ALIX and TSG101, along with the lack of contamination from common co‐isolates, lipoproteins and cellular membrane fractions like the endoplasmic reticulum and Golgi apparatus (Thery et al., 2018).

Regarding the mechanism of action of TDO2‐EVs, the authors conducted solid validations on how treatment with TDO2‐CCM attenuates damage to cardiac tissues and suppresses the expression of genes involved in inflammatory activation and senescence (Ciullo et al., 2023). They carried out rigorous studies to demonstrate that the immunomodulatory effects of TDO2‐EVs are mainly attributable to EVs. Indeed, NT4 was shown to be enriched in the EV fraction and deficient in the EV‐depleted fraction by size exclusion chromatography and qPCR. Furthermore, silencing NT4 in TDO2‐CCM abrogated the immunomodulatory effects. However, there is still a possibility that other components present in the TDO2‐CCM may also contribute to the observed cardioprotective functions and anti‐inflammatory effects. Non‐EV‐associated secretome present in the CCM may unexpectedly induce phenotypic changes in recipient cells. Additional experiments to compare the effects of isolated TDO2‐EVs and TDO2‐CCM would be informative to account for the relative contribution of EVs and non‐EV components to the cardioprotective effect of TDO2‐CCM.

Monocyte‐derived macrophages have been established as primary targets of cardioprotective CDC‐EVs (de Couto et al., 2017, 2015,2017, 2015, 2019). For that reason, the authors sought to examine immunomodulation of macrophages by NT4 in greater detail. Bone marrow‐derived macrophages were transfected with NT4, an NT4 scramble control, or TDO2‐CCM containing an antisense transcript to NT4 (Ciullo et al., 2023). The macrophages were stimulated with lipopolysaccharide (LPS) before being lysed for analysis of cytokine expression. Treatment with both NT4 and TDO2‐CCM was found to reduce expression of inflammatory cytokines IL‐1β, TNF‐α, and the monocyte recruitment chemokines CXCL1, 2, and 3. Compared to other treatment groups, NT4 and TDO2‐CCM also increased expression of pro‐angiogenic VEGF. Macrophages exposed to TDO2‐CCM with abolished NT4 function did not exhibit this reduced inflammatory or improved angiogenic profile. It is noteworthy that cardiac macrophages are heterogenous and elicit both overlapping and distinct functions (Duncan et al., 2020). Macrophages expressing high levels of MHC‐II are efficient at antigen presentation, whereas those expressing low levels of MHC‐II display strong phagocytic capacity (Epelman et al., 2014). CCR2^+^ macrophages are enriched in genes regulating the NLPR3 inflammasome pathway. Resident cardiac macrophages may also behave differently from peripheral and bone marrow‐derived macrophages. Given the important regulatory role of macrophages in cardiac remodelling, it is of interest to determine how TDO2‐EVs modulate the functions of different macrophage subsets, and which subsets, upon targeting, would produce the greatest benefits to tissue repair. ‘Indeed, this represents an active area of investigation by our group. Approaches like single‐cell sequencing (ranging from early to late time points post‐administration in an injury model) will be the key in uncovering the signalling trajectory of NT4 in circulating and resident macrophages and the predominant phenotype that NT4 promotes to drive tissue repair,’ says Ibrahim.

On another note, the authors of the study used lentiviral vectors (LVVs) to induce TDO2 overexpression in human dermal fibroblasts (Ciullo et al., 2023). While a highly stable and efficient transduction system, the concomitant risks of LVVs limit clinical translatability of the findings. Lentiviral vectors are characterized by stable integration into the genomes of target cells. The mechanisms of potential insertional mutagenesis and oncogenicity of LVVs are well‐documented (Schlimgen et al., 2016). Some LVVs contain accessory virulence factors (Milone & O'Doherty, 2018) which may be poorly tolerated in clinical translation or hamper therapeutic effects. In the current study, it is undetermined whether TDO2‐EVs contain remnants of LV transduction in the form of immunogenic viral RNAs. Thus, for clinical applications, ‘EV therapy generated by cells transduced with LVVs should be rigorously investigated for contamination of viral remnants,’ explains Ibrahim. Furthermore, the safety of therapeutic platforms using LVVs may be improved with third‐generation, self‐inactivating vectors, which have been investigated in several clinical trials (Milone & O'Doherty, 2018; Modlich et al., 2009). These LVVs are composed of viral genomes split into separate plasmids and an altered 3′ long terminal repeat sequence to prevent recombination. This new generation of LVVs may reduce the risk of mutagenesis, although their long‐term safety and efficacy still need to be assessed.

In summary, the study by Ciullo et al. presents the mechanism by which TDO2‐CCM attenuates inflammatory activation to minimize tissue damages post‐MI (Ciullo et al., 2023). This work is a continuation of the mechanistic dissection of the role of canonical Wnt signalling in cell and EV therapeutic potency. This is a highly commendable work which provides valuable information on molecular determinants of the potency of TDO2‐engineered cells and their EVs for the treatment of heart injuries and beyond. Despite the aforementioned limitations, the results are promising: this platform has great potential in regenerative medicine, warranting further investigations in future work.

## AUTHOR CONTRIBUTIONS

**Phuong H.D. Nguyen**: Conceptualisation; writing—original draft; writing—review & editing. **Clarissa Sastrawidjaya**: Visualization; writing—original draft; writing—review & editing.

## CONFLICT OF INTEREST STATEMENT

The authors declare no conflict of interest.
